# Case Report: Failed response to anti-PD-1 immunotherapy in a colon cancer patient with high microsatellite instability

**DOI:** 10.3389/fonc.2025.1636122

**Published:** 2025-10-07

**Authors:** Rong Li, Yan-Ping Lin, Xin Shen, Jiang-Yan Guo, Ya Zhang, Jia-Dai Tang, Lin Xie, Feng-Di Hu

**Affiliations:** ^1^ Department of Digestive Neoplasms, The Third Affiliated Hospital of Kunming Medical University, Yunnan Cancer Hospital, Peking University Cancer Hospital Yunnan, Kunming, Yunnan, China; ^2^ Department of Pathology, The Third Affiliated Hospital of Kunming Medical University, Yunnan Cancer Hospital, Peking University Cancer Hospital Yunnan, Kunming, Yunnan, China; ^3^ Department of Radiology, The Third Affiliated Hospital of Kunming Medical University, Yunnan Cancer Hospital, Peking University Cancer Hospital Yunnan, Yunnan Cancer Hospital, Kunming, Yunnan, China

**Keywords:** anti-PD-1 immunotherapy, microsatellite instability-high (MSI-H), colorectal cancer (CRC), immune resistance, biomarkers, case report

## Abstract

**Background:**

Although immune checkpoint inhibitors (ICIs) have achieved remarkable progress in the treatment of deficient mismatch repair (dMMR)/high microsatellite instability (MSI-H) colorectal cancer (CRC), nearly 50% of dMMR/MSI-H CRC patients exhibit primary resistance to immunotherapy.

**Case summary:**

An 84-year-old male patient was diagnosed with poorly differentiated adenocarcinoma of the right hemicolon (pT3N2M1c, stage IVc, dMMR). The patient underwent palliative surgery of the right hemicolon and subsequently received 3 cycles of bevacizumab in combination with capecitabine. Genetic testing revealed MSI-H/TMB-H/HLA heterozygosity. When the patient came to our center for treatment, we adjusted the treatment regimen to tislelizumab immunotherapy for 4 cycles. After immunotherapy, a CT review revealed disease progression. Moreover, the patient’s physical strength deteriorated dramatically, with an Eastern Cooperative Oncology Group (ECOG) score of 3. The patient subsequently received best supportive care. The patient’s overall survival (OS) was 9 months.

**Conclusion:**

The continued success of immunotherapy in dMMR/MSI-H CRC faces challenges related to immune resistance. Further studies are needed to uncover the mechanisms, targets, and biomarkers involved.

## Introduction

Colorectal cancer (CRC) ranks as the third most commonly diagnosed malignancy worldwide (after breast and lung cancers) and represents the second leading cause of cancer-related mortality (1). In China, CRC ranks second in incidence rate and fourth in mortality rate among malignant tumors, with the incidence rate increasing annually (2). Defects in the function of mismatch repair genes lead to deficient mismatch repair (dMMR), resulting in the improper repair of randomly generated errors during DNA replication, which leads to high microsatellite instability (MSI-H) (3). MSI-H occurs in 19.7% of colon adenocarcinoma patients and 5.7% of rectal adenocarcinoma patients (4). Research on immune checkpoint inhibitors (ICIs) for the treatment of solid tumors, including the results of KEYNOTE-016 (5), CheckMate 142 (6), KEYNOTE-158 (7), and KEYNOTE-177 (8), has gradually revealed that patients with dMMR/MSI-H metastatic colorectal cancer constitute a favorable population for immunotherapy. Guidelines from the NCCN, ESMO, and other expert consensuses recommend the use of ICIs as the first-line treatment for dMMR/MSI-H metastatic colorectal cancer (mCRC).

Currently, ICIs have achieved breakthrough success in both palliative and perioperative treatment of dMMR/MSI-H CRC. However, In both clinical studies and clinical practice, we still observe that immunotherapy is ineffective in nearly 50% of dMMR/MSI-H CRC patients (9), which indicates a need to further explore the mechanisms of drug resistance in dMMR/MSI-H CRC patients, identify more clinically significant indicators to predict the efficacy of immunotherapy, and accurately screen the population that will benefit from immunotherapy. In this article, we report a case of dMMR/MSI-H/tumor mutational burden-high (TMB-H) metastatic bowel cancer in which anti-PD-1 therapy was ineffective, and we explore the possible mechanisms of resistance in this patient and similar cases by analyzing their pathological features and gene expression status, while also reviewing the relevant literature.

## Case presentation

### Chief complaints

An 84-year-old elderly Chinese male presented to our hospital for the first time in March 2022, 3 months after right hemicolectomy and 3 weeks after his last chemotherapy session.

### History of present illness

The patient was admitted to a hospital in Yunnan Province, China, with a one-month history of hematochezia and abdominal pain. Over the previous four days, he developed symptoms of anal obstruction and inability to pass flatus. Following comprehensive examinations, the patient was diagnosed with right-sided colon cancer. On November 29, 2021, he underwent a right hemicolectomy and experienced an uneventful recovery. Six weeks postsurgery, he received three cycles of combination therapy with bevacizumab and capecitabine.

### Baseline demographic and clinical characteristics

The patient, with a body mass index (BMI) of 21.37 kg/m², had a medical history of hypertension for 30 years. His highest recorded blood pressure was 150/90 mmHg, which was well-controlled with daily eprosartan (600 mg). He had also undergone a cholecystectomy 16 years earlier. There was no history of diabetes, cardiovascular disease, or chronic kidney disease. His Eastern Cooperative Oncology Group (ECOG) performance status was 0 (fully active) before diagnosis but declined to ECOG 1 at the time of presentation.

### Social history

The patient denied a family history of tumors and reported no smoking or alcohol consumption.

### Physical examination

ECOG performance status: 1. Vital signs included a temperature of 36.5 °C, blood pressure of 138/89 mmHg, heart rate of 75 beats per minute, and respiratory rate of 19 breaths per minute. There is a 15 cm longitudinal surgical scar in the midline of the abdomen and an older scar approximately 10 cm in length below the right costal margin. The abdomen was soft and nontender.

### Laboratory examinations

**Figure 1:**
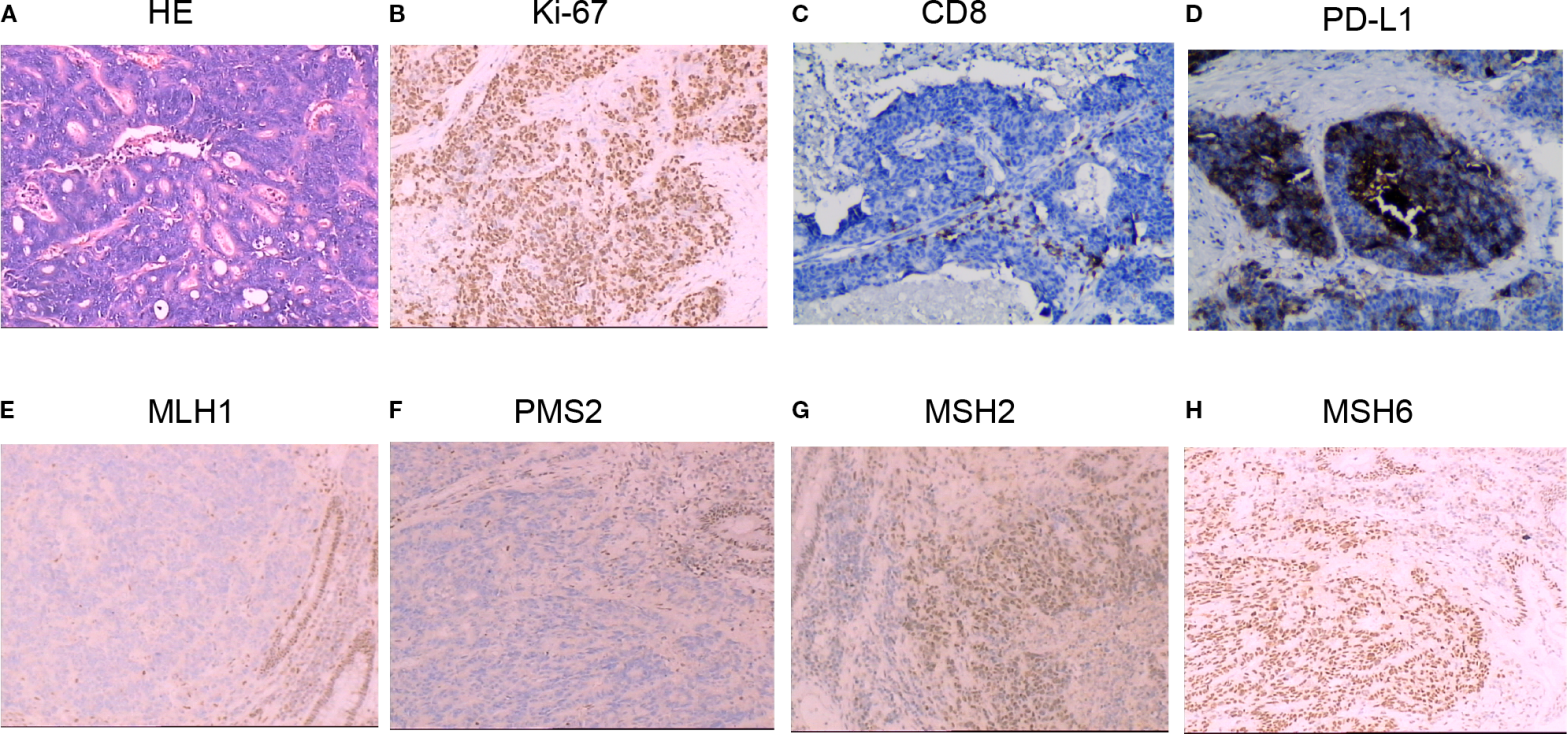
Haematoxylin plus eosin (H&E) and immunohistochemistry (IHC) staining of colon cancer tissue samples. **(A)** Pathologic findings indicating poorly differentiated adenocarcinoma post-colon surgery. **(B)** Immunohistochemical results suggesting 80% ki-67-positive cells. **(C)** Sparse infiltration of CD8+ T lymphocytes (<50 cells/mm²). **(D)** PD-L1 expression with CPS = 20. **(E–H)** Mismatch repair (MMR) expression results by immunohistochemistry, revealing MLH1 (-), PMS2 (-), MSH2 (+), and MSH6 (+).

Postoperative pathology revealed an 8 cm poorly differentiated adenocarcinoma tumor (**Figure 1A**) with infiltration into the fibroadipose tissue beyond the intrinsic muscularis propria. Cancer metastasis was observed in 3 out of 14 lymph nodes, accompanied by 2 cancerous nodules. The immunohistochemistry (IHC) results revealed the following: Ki67: 80% (**Figure 1B**), CD8+ T lymphocytes (<50 cells/mm²) (**Figure 1C**). Diffuse PD-L1 expression on tumor cells and immune cells with Combined Positive Score (CPS) = 20 (**Figure 1D**). MLH1 (-), PMS2 (-), MSH2 (+), and MSH6 (+) (**Figures 1E–H**).

Next-generation sequencing (NGS) analysis of the patient’s postoperative tissue paraffin sections and peripheral blood ctDNA revealed MSI-H, TMB-H (86.59 Muts/Mb), HLA heterozygosity, wild-type KRAS/NRAS/BRAF, NTRK1 rearrangements, and mutations in PTEN, TP53, and CDH1, among others (**Table 1**).

**Table 1:** Results of sequence analyses of paraffin sections and blood cells.

Gene	Tissue		Blood	
	Mutation site	Mutation abundance (paraffin sections)	Mutation site	Mutation abundance (ctDNA)
TP53	p.R282W Exon 8	32.31%	p.R282W c.844 C>T	32.69%
	p.P72Afs*51 Exon 4	50.02%	p.P72Afs*51 c.214-215delinsG	27%
PTEN	p.P96Q Exon 5	35.34%	p.P96Q c.287C>A	27.03%
APC			p.R499Q c.146G>A	27.30%
CDH1	p.R598* Exon 12	34.22%		
ARID1A	p.P1326fs*155 Exon 16	34.33%		
NTRK1	TPM3-NTRK1(E1-7;E9-17) rearrangements	39.68%		
	TPM3-NTRK1(E1-7;E9-17) rearrangements	35.61%		
MLH1	p.R659* Exon 17	39.57%		
PMS2	p.D414RFs*44 Exon 11	31.34%		
TBM		86.59Muts/Mb		
HLA		heterozygosis		

**Figure 2:**
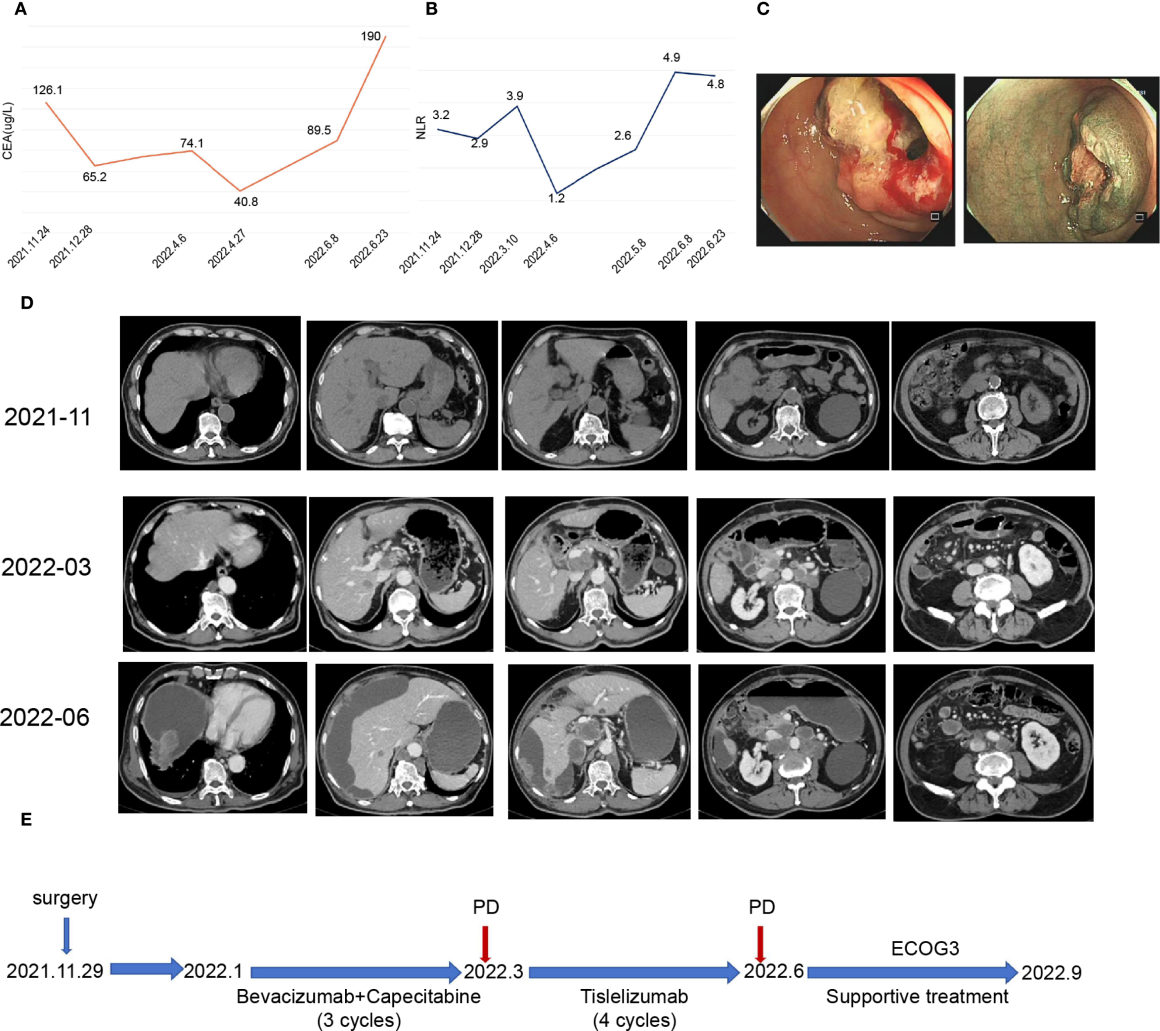
Patient clinical data and medical treatment history. **(A)** Plot showing changes in carcinoembryonic antigen (CEA) levels during treatment. **(B)** Graph illustrating changes in the neutrophil–to-lymphocyte ratio (NLR) during treatment. **(C)** Enteroscopy revealed cauliflower-like growth located 60 cm from the anal opening, which encircled the lumen and resulted in luminal stenosis. **(D)** Representative imaging images depicting the treatment process. **(E)** Timeline of the patient treatment process.

On November 24, 2021, the carcinoembryonic antigen (CEA) level was elevated to 126.1 µg/L (**Figure 2A**), and the neutrophil-to-lymphocyte ratio (NLR) was measured at 3.2 (**Figure 2B**).

### Imaging examinations

Enteroscopy revealed a cauliflower-like growth located 60 cm from the anal verge. This growth encircled the lumen, causing luminal narrowing and preventing the passage of the CF-H290I endoscopic instrument (**Figure 2C**).

CT examination revealed a malignant tumor located at the hepatic flexure of the colon, accompanied by stenosis and multiple lymph node metastases within the peritoneum and abdominal cavity (**Figure 2D**).

## Final diagnosis

Based on the patient’s medical history, the final diagnosis was poorly differentiated adenocarcinoma of the right-sided colon, with a pathological stage of pT3N2M1c (clinical stage IVc).

## Treatment

In March 2022, the patient presented with Grade III hand–foot syndrome and Grade II myelosuppression. Immunohistochemical and genetic testing revealed dMMR/MSI-H status. Consequently, the treatment plan was adjusted to include tislelizumab immunotherapy (200 mg, every 3 weeks).

### Detailed timeline of diagnostic and therapeutic decisions

November 2021:

Admission to local hospital following 4 days of complete obstruction.

Emergency enteroscopy revealed obstructing right colon mass.

CT scan confirmed hepatic flexure tumor with peritoneal metastases.

Decision rationale: Surgical intervention indicated due to complete bowel obstruction.

Right hemicolectomy performed on November 29, 2021.

January 2022:

Decision rationale: Based on the stage IV diagnosis and relatively preserved functional status (ECOG 1), first-line chemotherapy with bevacizumab plus capecitabine was initiated. The patient received three cycles administered every three weeks.

March 2022:

Presented to our institution with Grade 3 hand-foot syndrome and Grade 2 myelosuppression.

Decision rationale: Based on dMMR/MSI-H status and treatment-related toxicity, decided to switch to immunotherapy.

Initiated tislelizumab 200 mg every 3 weeks.

## Outcome and follow-up

Throughout the treatment period, the patient’s CEA levels fluctuated with disease progression (**Figure 2A**), while the NLR remained almost above 3 (**Figure 2B**). Despite undergoing 4 cycles of immunotherapy, follow-up CT scans in June 2022 indicated disease progression (**Figure 2D**). The patient’s physical condition deteriorated significantly, resulting in an ECOG score of 3. The patient’s physical condition deteriorated significantly, with his ECOG performance status declining to 3, necessitating best supportive care: thoracentesis was performed to manage malignant pleural effusion; enteral and parenteral nutrition were initiated to maintain adequate caloric intake; and comprehensive pain control was implemented according to the WHO analgesic ladder protocol. Despite receiving optimal supportive care, the patient ultimately succumbed to the disease in September 2022, with an overall survival duration of nine months from the initial diagnosis (**Figure 2E**).

## Discussion

This case report details a colon cancer patient exhibiting MSI-H/TMB-H features and HLA heterozygosity, conditions that would typically suggest sensitivity to immune checkpoint inhibitors based on their genomic profile. However, the patient showed rapid disease progression and a dramatic deterioration in physical status after PD-1 inhibitor therapy. This unexpected response prompted an in-depth investigation into potential resistance mechanisms.

### Analysis of high-risk factors at baseline

The 84-year-old patient exhibited several poor prognostic features: poorly differentiated histology (Ki-67 index of 80%), indicative of highly aggressive tumor biology. Notably, although TMB-H generally predicts chemotherapy sensitivity, the patient exhibited primary resistance during first-line bevacizumab plus capecitabine therapy, as evidenced by persistently elevated CEA levels. PTEN mutation may reduce chemotherapeutic efficacy by impairing the DNA damage response via AKT pathway activation (10). The decision to withhold first-line immunotherapy was likely based on limited safety data in octogenarians and local insurance restrictions. Potential mechanisms contributing to second-line immunotherapy failure include:

1. Age-related T-cell dysfunction: Senescence-associated impairment of CD8+ tissue-resident memory T cells could compromise anti-tumor immune responses (11).
2. Systemic inflammation: Persistently elevated neutrophil-to-lymphocyte ratio (NLR≥3) during treatment correlates with poor immunotherapy outcomes, possibly via CD80/CD86-CTLA4 axis-mediated immunosuppression (12, 13).

### Genetic mutations and tumor microenvironment features

Genomic testing identified mutations in PTEN, CDH1, and APC, although the lack of protein expression validation remains a study limitation. Despite high PD-L1 expression, the tumor microenvironment showed minimal CD8+ T-cell infiltration. Existing literature indicates these mutations could disrupt immune cell subsets—such as Tregs, MDSCs, and TAMs—via intricate signaling networks, fostering an immunosuppressive environment that hinders CD8+ T-cell activity. For instance, PTEN mutations are linked to diminished PD-1 inhibitor responses in dMMR/MSI-H gastrointestinal cancers, likely due to PI3K/AKT/mTOR pathway activation and CD8+ T-cell depletion (14, 15). Similarly, CDH1 mutations might facilitate immune evasion by increasing CD56 bright NK cell accumulation (9), while APC loss could reduce immunotherapy sensitivity through β-catenin/TCF4-PD-L1 axis activation and WNT pathway dysregulation (16–18). These findings highlight potential mechanisms underlying the observed immune evasion and therapeutic resistance.

### Clinical implications of NTRK fusion

The co-occurrence of NTRK fusion with MSI-H aligns with the reported characteristics of NTRK-rearranged colorectal cancers (typically BRAF/RAS wild-type) (19, 20). While targeted therapy (e.g., entrectinib) offers a potential salvage option (21), the patient’s rapid decline in ECOG performance status precluded such an intervention. Notably, the prevalence of NTRK fusion is 1-5% in dMMR CRC, particularly in those with MLH1/MSH2 deficiency (19), though its relationship with immunotherapy resistance requires further investigation.

This case highlights the need for caution regarding primary resistance in high-risk dMMR/MSI-H patients (poor differentiation, peritoneal metastasis, NLR≥3) even with TMB-H/HLA heterozygosity. Potential strategies include:

1. Combination Therapy with ICIs.

Combining different ICIs is a promising strategy to overcome immune resistance in patients with bowel cancer. According to 5-year follow-up data from the CheckMate 142 study, the combination of nivolumab (NIVO) and ibritumomab is recommended across all treatment lines for patients with dMMR/MSI-H advanced bowel cancer (6). The CheckMate 8HW trial demonstrated superior efficacy of NIVO plus ipilimumab (IPI) compared to NIVO monotherapy in MSI-H/dMMR metastatic colorectal cancer (CRC) patients. This combination not only significantly prolonged progression-free survival (PFS) but also yielded durable responses in long-term follow-up, suggesting an early potential to overcome resistance to single-agent immunotherapy (22). LAG-3, which inhibits T-cell proliferation and cytokine production, promotes tumor escape through multiple mechanisms (23). Dual inhibition of PD-1 and LAG-3 is thought to enhance immune responses in patients resistant to anti-PD-1/PD-L1 therapies (24). In 2022, the FDA approved the combination of nivolumab and relatlimab (a LAG-3 inhibitor) for treating unresectable or metastatic melanoma (23). Additionally, the NICHE-3 study presented at the 2023 ESMO Congress demonstrated, for the first time, the efficacy and safety of nivolumab combined with relatlimab in dMMR colon cancer, with an impressive overall pathological remission rate of 100% (25).

2. Novel Target-Based Immunotherapies.

Guanylate cyclase C (GCC) is expressed in approximately 95% of colorectal cancer tumors and serves as a specific tumor marker for CRC (26). CAR-T-cell therapies targeting GCC have shown definitive efficacy in animal models of metastatic colorectal cancer (27). A recent Phase I clinical trial reported that IM96, a CAR-T-cell therapy targeting GCC, achieved a disease control rate (DCR) of 66.7% in patients with GCC-positive metastatic CRC who had failed at least three prior therapies. Tumor remission in responders lasted over 9 months (28). Transforming growth factor-β (TGF-β) inhibits CD8+ T-cell cytotoxicity by suppressing genes involved in T-cell lysis, such as perforin, granzymes A and B, and interferon-γ (IFN-γ) (29). Integrin αvβ6 is a major activator of TGF-β; its inhibition can block TGF-β activation, prevent immune evasion by tumors, and enhance the efficacy of immune checkpoint blockade therapy, making it a promising therapeutic target in CRC (30). Furthermore, the discovery of new immune checkpoints, such as TIM-3, TIGIT, and B7-H3, may help overcome immune resistance in solid tumors (31).

3. Combination Therapy with ICIs and Antiangiogenic Agents.

Antiangiogenic therapies can enhance immunotherapy efficacy and inhibit tumor growth by promoting effector T-cell infiltration and reducing the levels of inhibitory elements in the tumor microenvironment (TME), such as Tregs, MDSCs, and M2-TAM (32). The REGONIVO study demonstrated that regorafenib, a small-molecule multitarget antiangiogenic drug, in combination with nivolumab, achieved an objective response rate (ORR) of 36% in patients with metastatic bowel cancer who had received more than three lines of treatment (33). Therefore, the combination of antiangiogenic drugs, which alter the TME, with immunotherapy presents a promising approach to overcome immune resistance.

4. Targeted Therapy.

Targeted agents directed against rare molecular alterations offer a viable option for patients with dMMR/MSI-H CRC who have failed immunotherapy. Notably, HER2- and NTRK-targeted therapies have shown promise. Emerging evidence suggests that CRC patients with both MSI-H and actionable gene mutations may represent a distinct molecular subtype of dMMR/MSI-H tumors (34). Multiple studies have reported HER2 amplification or overexpression in 2.2%–9.5% of primary CRC cases (35). The MOUNTAINEER trial demonstrated an objective response rate (ORR) of 38.1%, with 3 of 32 responders achieving complete remission. Based on these results, the FDA granted accelerated approval in January 2023 for tucatinib plus trastuzumab for RAS wild-type/HER2-positive unresectable or metastatic CRC progressing after at least one prior chemotherapy regimen (36). Additionally, antibody-drug conjugates (ADCs) like trastuzumab deruxtecan (T-DXd, DS-8201) have shown promising efficacy in later-line treatments for metastatic CRC, with an ORR of 45.3% (37). Clinical trials consistently demonstrate the remarkable efficacy of TRK inhibitors in advanced NTRK-rearranged tumors, regardless of primary site or histology. The FDA has approved both entrectinib and larotrectinib for the treatment of locally advanced or metastatic NTRK fusion-positive solid tumors (38).

## Conclusion

We present an 84-year-old patient with metastatic right-sided colon cancer exhibiting dMMR/MSI-H/TMB-H and HLA heterozygosity who showed primary resistance to anti-PD-1 immunotherapy despite favorable biomarkers, highlighting a significant paradox: while the tumor expressed high PD-L1 levels, it demonstrated extremely low CD8+ T-cell infiltration, indicating an immunologically “cold” tumor microenvironment. Although immunotherapy has transformed dMMR/MSI-H CRC treatment, our findings suggest that PTEN/CDH1/APC mutations, immune senescence linked to advanced age, and persistently elevated neutrophil-to-lymphocyte ratio (NLR > 3) may collectively drive primary resistance, emphasizing the need to integrate tumor mutation profiling, microenvironment analysis, and host factors to optimize treatment for high-risk subgroups.

This study has limitations, including unvalidated resistance mechanisms due to insufficient tissue for PTEN/CDH1/APC IHC confirmation, gaps in dynamic profiling from missing paired pre-/post-treatment specimens for genomic evolution (e.g., NTRK fusion clonality) and spatial TME analysis, and age-related constraints preventing further targeted therapies (e.g., entrectinib for NTRK fusion) or biopsies due to rapid clinical decline. Future research should focus on multi-omics integration (e.g., single-cell RNA-seq and TCR repertoire) to uncover CD8+ T-cell dysfunction mechanisms and develop clinically applicable predictive models combining mutation signatures (PTEN/CDH1/APC), CD8+ T-cell density, and systemic inflammation markers (e.g., NLR).

## Data Availability

The original contributions presented in the study are included in the article/supplementary material. Further inquiries can be directed to the corresponding authors.
